# Supplementary material for: Monitoring reproduction in cryptic small mammals; using body temperature to identify parturition in an endangered rodent
Source: Biol Open. 2026 Apr 28;15(4):bio062459. doi: 10.1242/bio.062459 (PMC13225210; doi:10.1242/bio.062459)
Supplement: Supplementary information [file biolopen-15-062459-s1.pdf]

Genetics

Table S1. Details of the analysed microsatellite loci.

		2022						2023						2024					
Loci	mix	k	n	HObs	HExp	PIC	F(Null)	k	n	HObs	HExp	PIC	F(Null)	k	n	HObs	HExp	PIC	F(Null)
IPK 01	1	6	363	0.807	0.722	0.674	-0.058	6	245	0.735	0.719	0.672	-0.015	6	195	0.749	0.738	0.700	-0.012
IPK 12	1	5	363	0.590	0.523	0.409	-0.062	2	245	0.498	0.498	0.373	-0.001	3	195	0.523	0.500	0.377	-0.023
IPK 05	1	4	363	0.471	0.453	0.401	-0.017	4	245	0.600	0.559	0.479	-0.036	5	195	0.451	0.431	0.395	-0.031
IPK 07	1	7	363	0.741	0.677	0.636	-0.044	7	244	0.803	0.696	0.640	-0.080	5	195	0.754	0.679	0.627	-0.051
IPK 09	1	5	363	0.763	0.693	0.640	-0.054	4	245	0.735	0.685	0.630	-0.039	4	195	0.708	0.646	0.594	-0.052
IPK 06	1 bis	4	363	0.639	0.543	0.490	-0.088	4	216	0.611	0.580	0.525	-0.027	x	x	x	x	x	x
Ccrµ 15	2	4	364	0.681	0.633	0.555	-0.040	5	246	0.593	0.620	0.540	0.025	3	194	0.552	0.589	0.513	0.027
Ccrµ 3	2	5	364	0.536	0.528	0.465	-0.012	4	246	0.581	0.561	0.488	-0.016	4	194	0.639	0.624	0.567	-0.014
Ccrµ 20	2	6	364	0.706	0.619	0.568	-0.078	5	245	0.567	0.553	0.514	-0.011	5	195	0.733	0.715	0.669	-0.011
Ccrµ 10	2	3	364	0.679	0.646	0.569	-0.026	3	246	0.610	0.576	0.485	-0.030	3	195	0.564	0.586	0.499	0.017
Ccrµ 11	3	7	363	0.749	0.749	0.707	-0.025	8	246	0.740	0.720	0.671	-0.014	6	194	0.675	0.656	0.602	-0.013
Ccrµ 4	3	2	363	0.515	0.494	0.372	-0.022	3	245	0.551	0.478	0.365	-0.072	2	195	0.579	0.496	0.372	-0.079
IPK 02	3	4	364	0.690	0.688	0.630	0.002	4	247	0.798	0.719	0.665	-0.055	5	194	0.773	0.737	0.686	-0.028
Ccrµ 17	3	3	364	0.143	0.135	0.129	-0.028	2	247	0.081	0.078	0.075	-0.012	3	194	0.258	0.247	0.228	-0.033
Ccrµ 19	3	3	364	0.544	0.473	0.391	-0.073	4	247	0.559	0.518	0.446	-0.037	4	195	0.595	0.562	0.467	-0.027

Statistics

Table S2. Statistical outputs of the models investigating the link between T°b metrics and females Status (LMM models with the female ID as a random factor). Pairwise comparisons among status are presented in Table S3.

Investigated T°b metric:	µT°b		ΔT°b		T°b SD		βT°b pre		βT°b post	
Model p-value	< 0.001		< 0.001		< 0.001		< 0.001		< 0.001	
Statistics	β	t	β	t	β	t	β	t	β	t
Intercept (Parturition)	38.815	316.452	0.906	19.660	0.259	7.508	-0.053	-3.156	-0.012	-0.411
None	-0.701	-6.117	-0.922	-19.080	0.067	1.931	0.056	3.190	0.040	1.378
End of Gestation	-1.461	-13.267	-0.921	-19.880	0.255	7.683	0.046	2.684	0.016	0.560
Rearing Pups	-0.768	-6.925	-0.952	-20.360	0.126	3.780	0.034	1.978	-0.052	-1.870
With Male	-0.890	-8.000	-0.873	-18.630	0.205	6.121	0.089	5.191	-0.003	-0.102

Table S3. Outputs of the Tukey tests underling Table 1. Pairwise comparisons of daily T°b metrics for the different females' status, extracted from the LMM models with the female ID as a random factor. Each column presents the Tukey test estimates and p-value. Statistically significant differences (Tukey, $p < 0.05$) are indicated in bold. n = 2245 days.

Investigated T°b metric:	µT°b			ΔT°b			T°b SD			βT°b pre			βT°b post		
Status pairs comparisons	β	z	p-value	β	z	p-value	β	z	p-value	β	z	p-value	β	z	p-value
Parturition Day - None	**-1.461**	**-13.267**	**<0.001**	**-0.922**	**-19.875**	**<0.001**	**0.255**	**7.683**	**<0.001**	**0.046**	**2.684**	**0.046**	0.016	0.560	0.977
Parturition Day - Male presence	**-0.890**	**-8.000**	**<0.001**	**-0.873**	**-18.634**	**<0.001**	**0.205**	**6.121**	**<0.001**	**0.0890**	**5.191**	**<0.001**	-0.003	-0.102	1.000
Parturition Day - Gestating	**-0.701**	**-6.117**	**<0.001**	**-0.922**	**-19.076**	**<0.001**	0.067	1.931	0.266	**0.056**	**3.190**	**0.010**	0.040	1.378	0.606
Parturition Day - Rearing Pups	**-0.768**	**-6.925**	**<0.001**	**-0.952**	**-20.359**	**<0.001**	**0.126**	**3.780**	**0.001**	0.034	1.978	0.242	-0.052	-1.870	0.298
Male presence - None	**0.572**	**23.116**	**<0.001**	**0.048**	**4.765**	**<0.001**	**-0.050**	**-6.703**	**<0.001**	**0.043**	**11.531**	**<0.001**	**-0.019**	**-2.947**	**0.021**
Gestating - None	**-0.761**	**-20.305**	**<0.001**	0.001	0.011	1.000	**0.188**	**16.694**	**<0.001**	-0.011	-1.892	0.285	-0.024	-2.566	0.063
Rearing Pups - None	**0.693**	**28.497**	**<0.001**	**-0.031**	**-3.190**	**0.010**	**-0.129**	**-17.591**	**<0.001**	**-0.012**	**-3.222**	**0.009**	**-0.068**	**-10.769**	**<0.001**
Gestating - Male presence	**-0.189**	**-4.676**	**<0.001**	**0.048**	**2.854**	**0.028**	**0.138**	**11.357**	**<0.001**	**0.033**	**5.261**	**<0.001**	**-0.043**	**-4.187**	**<0.001**
Rearing Pups - Male presence	**-0.122**	**-4.232**	**<0.001**	**0.079**	**6.662**	**<0.001**	**0.079**	**9.092**	**<0.001**	**0.055**	**12.654**	**<0.001**	**0.050**	**6.704**	**<0.001**
Rearing Pups - Gestating	-0.067	-1.703	0.393	-0.031	-1.842	0.311	**0.060**	**5.000**	**<0.001**	**-0.023**	**-3.688**	**0.002**	**-0.093**	**-9.132**	**<0.001**

Biology Open • Supplementary information
